# Retrospect of the Medical Sciences

**Published:** 1843-03-04

**Authors:** 


### SUCCESSFUL OPERATION FOR STRANGULATED HERNIA, AT THE AGE OF 107!

We do not know that there is any record of the performance of a severe operation at the advanced age of 107, which has, however, been performed by Mr. Cæsar Hawkins, with success, in St. George's Hospital; and we apprehend there is no doubt of the patient's real age, as the certificate of his birth has been preserved by his friends, one of whom came with him to the hospital, and asserted that he had seen it. The patient is, moreover, well known as a musician, who had the honour, as he expresses it, of playing on the violin with his late Majesty, George the Fourth. His name is Rochard, a Frenchman, and his faculties are quite entire; he was able to walk into the ward, and within these few days was strong enough to walk from Bayswater to Charing Cross, and back again.

This man came to the hospital about one o'clock on Thursday last (Nov. 24th) with strangulated inguinal hernia, which had been fixed from, at least, the previous morning, and perhaps for a longer time, as no motion had passed for three or four days : the tumour was hard and tender, and vomiting had repeatedly taken place, but the countenance was cheerful, and the pulse unaffected.

While some other operations were being performed, Mr. Hawkins informed the students of the case having been admitted, and said that two attempts to reduce the hernia had been made before his admission ; that the muscles being at his age completely relaxed, the only remedy likely to be useful was the application of ice, which he thought not unlikely to diminish the size of the tumour enough to enable it to recede, and that as the symptoms were not severe, and were generally slow in their progress at an advanced period of life, he would give him this chance, and see him again at five o'clock. When Mr. Hawkins came, however, at this period, he found that although there had been no sickness, yet the tumour was rather more tender, and therefore proceeded at once to the operation. The sac was very thick, and on opening it a mass of apparently transparent jelly protruded, which was a considerable quantity of recent lymph, distended with the serum, which filled the sac : the contents were a few inches of inflamed small intestine glued together and to the sac by lymph, which Mr. Hawkins was obliged, after dividing the stricture, to tear away from the bowel in order to reduce it. The case went on very well afterwards, and requires no particular notice;—evacuations were passed the next day, after an injection ;—some slight tenderness of the abdomen was relieved by chamomile poultice, and by one or two doses of the calomel on the Saturday. The wound united entirely by the first intention, except where two ligatures had been applied to a divided vessel of the outside of the sac, which part was slightly opened by the probe: the last section was removed on Sunday, and on Wednesday, the 30th, we saw him sitting up in a chair. We may observe that nourishment was given him from the first, and a little wine the last day or two ; he is rather weaker since the strangulation, but appears to be going on quite satisfactorily.

Dec. 7.—Continues to go on favourably in every respect ; in fact, he may be regarded as having entirely recovered.—*Lond. Med. Gaz.* Dec. 9.

### SUETTE MILIARE.

Five of the departments of France, in different parts of the kingdom, were visited during the past year with this pestiferous disease, the sweating-sickness of English authors, which caused such ravages in England in the fourteenth and fifteenth centuries, but which is now happily banished from this country, and is even rare among our neighbours. In one part of the department Dordogne, out of a population of 82,200 persons, 10,400 were attacked by the disease, of whom 800, or one in thirteen, perished. It was observed to rage mostly in marshy situations, and to be best treated in a manner similar to intermittent fever, viz., with quinine, and other tonics, taking particular care to interfere in no way with the march of the miliary eruption.—*Lond. Lan., from Gazette des Hopitaux.*

### PHYSIOLOGY OF MENSTRUATION.

Dr. Raciborski has communicated to the French Academy of Sciences the following conclusions which he has drawn from his researches on this subject. Very intimate relations exist between the catamenia and the Graafian vesicles ; menstruation begins at the period when these are fully developed, and ceases whon they are effete (detruits.) At each menstrual period one of these vesicles swells and projects from the surface of the ovary, from which it escapes by the rupture of its sac, usually about the end of the menstrual flow, without male congress or other sexual excitement. The vesicle which thus escapes has anatomical characters precisely similar to those of the corpus luteum supposed to be formed after conception. Diseases arrest the developement of the vesicles, and it is in this arrest that the true cause of amenorrhœa is in many cases to be sought ; for the catamenial flow is the result of the sanguineous congestion of the internal generative organs, by which the developement of the vesicle in its highest degree is accompanied. The state of the ovaries is so intimately connected with the general health of an individual, that the internal appearance of these organs after death is said by Raciborski to be alone adequate to determine whether the patient has sunk under an acute or a chronic disease, or whether she has latterly menstruated regularly.

Phenomena analogous to those specified above as occurring in the human female at the menstrual period, are remarked also in animals during their rutting season. Among these the ovarian vesicles are found to increase gradually in size during the interval between the rutting epochs ; and they ultimately escape altogether at these epochs without congress of the male. The spontaneous detachment of the human ovum at the end of the menstrual epoch naturally renders that period the most favourable for impregnation, and readily yields a reason for the fact that conception is most commonly referred to that epoch by pregnant women.

" Of 15 women (says Dr. R.) who specified accurately the period of their latest menstruation, as well as the dates of the connubial act, 5 evidently conceived from coitus taking place from two to four days previous to the period at which the catamenia was due. In 7, conception dated from coitus occurring two or three days after menstruation ; in 2, it took place at the actual period of the catamenia ; and in one only so long as ten days after the latter had disappeared."

Considered with respect to her generative function, woman holds a place intermediate between rutting animals, which are capable of impregnation only at fixed seasons of the year, and those animals in which a coitus only is required to produce impregnation at any season. She, however, approaches much nearer in point of this analogy to the former class, her power of reproduction being infinitely the more active at her menstrual periods, to which the rutting time in brutes bears a strict physiological resemblance.—*Gaz. des Hopitaux.*

There is much that is new, rational, and striking in the above views, and we shall keep an eye upon any future observations that may be made by Dr. Raciborski on this subject.—*London Lancet*, January 28, 1843.

### THE THEORY OF CELLS OPPOSED.

*The Structure of Fibrinous Exudations, or False Membranes; Origin of Fibre.*—It has been commonly supposed that fibrine exhibits an organized appearance only when it has coagulated in contact with the living textures. In his Notes and Appendix to "Gerber's Anatomy," Mr. Gulliver has depicted a distinct structure in fibrine which has set either within or out of the body, simply from *rest;* and a similar character is shown in a false membrane. He now gives several more figures to exhibit the analogy, in structure, of fibrine coagulated merely from rest, and fibrinous exudations resulting from *inflammation.* This structure is made up of fibrils of extreme delicacy and tenacity, and of corpuscles possessing the character of primary cells, or organic germs.

Of late years the origin of fibre, as well as of all other tissues, has been ascribed to the growth of cells; but these observations of Mr. Gulliver render it probable that cells are not essential to the formation of all textures, since it would appear that fibrils, which may be the primordial fibres of certain parts, are formed, in a few minutes, by the simple act of *coagulation* in fibrine.

M. Gerber (Gen. Anat., figs. 16—18,) has delineated what he terms the first, the second, and the complete stages of *fibrillation*, in the progress of organization in the fibrine composing coagulable lymph; but he does not say how much his drawings are magnified, though in some of them a very low power must have been employed. Others are sufficiently enlarged to show the cells from which he says the fibres are formed, and this is precisely the point on which Mr. Gulliver says that his observations are at issue with the views now generally entertained concerning the origin of fibres.

"All the organic tissues," says Dr. Schwann, "however different they may be, have one common principle of developement as their basis, viz., the formation of *cells;* that is to say, nature never unites molecules immediately into a fibre, a tube, and so forth, and she always, in the first instance, forms a round cell, or changes, when it is requisite, the cells into various primary tissues, as they present themselves in the adult state." (Wagner's Physiology, by Willis, p. 222.)

"How," continues Mr. Gulliver, "is the origin of the fibrils which I have depicted in so many varieties of fibrine to be reconciled with this doctrine? And what is the proof that these fibrils may not be the primordial fibres of animal textures? I could never see any satisfactory evidence that the fibrils of fibrine are *changed cells;* and, indeed, in many cases the fibrils are formed so *quickly* after coagulation, that their production, according to the views of the eminent physiologist just quoted, would hardly seem possible; nor have I been able to see that these fibrils arise from the interior of the blood-discs, like certain fibres delineated in the last ingenious researches of Dr. Barry." Such are the remarks of Mr. Gulliver in his "Contributions to Minute Anatomy," in the number of the "Lond. and Edinb. Philos. Magazine," for Oct. 1842.—*Lond. Lancet*, Jan. 14, 1843.

### LACTUCARIUM.

A medical man belonging to the nation which has endeavoured to substitute the product of beet-root for the juice of the sugar-cane, has lately manifested considerable anxiety to introduce lactucarium into extensive use in pharmacy. It is, as may be known already, the concrete juice of several kinds of lettuce, and, in the opinion of M. Aubergier, ought to rival opium in medical practice, M. Aubergier announcing his hope that its employment in France will diminish the consumption of a production for which so high a price is paid "to foreigners." Lactucarium must be carefully distinguished from the watery extract of lettuce, in which it is contained only in a very small proportion, this proper juice of the plant being in an extremely small degree soluble in water, to which circumstance the great inferiority of narcotic power manifested by extract of lettuce is doubtless owing. Lactucarium is not present in all the species of *lactuca;* the *L. striata, acuminata, longata*, &c., yield only an insipid or sweetish juice, containing a great deal of mannite, but destitute of that principle on which the medicinal quality seems to depend. It is obtained in the greatest quantity from the *lactuca altissima*, which, by culture, may be made to reach ten feet in height, and from a few plantations of which gigantic vegetable, M. Aubergier calculates on obtaining a sufficient supply of the drug to expel the opium from the market of his country. The juice flows from a number of incisions made in the stalk and leaves at the time of inflorescence, and is at first of the colour and consistence of cream, but soon hardens, becoming successively of a yellow and a brown colour; and after having lost about 71 per cent. of its original weight, by evaporation, it presents itself covered with a crystalline efflorescence of mannite. Lactucarium contains a bitter principle, mannite, asparigin, a crystallisable matter (giving a green colour to the salts of iron,) a resin combined with potass, another resin, cerine, myricine, pectine, vegetable albumen, ulmate, oxalate, malate, nitrate, muriate and sulphate of potass, phosphate of lime and magnesia, oxides of iron and maganese, and silica. The bitter medicinal principle is a crystalline matter, which, as it is said to bear "the same relation to lactucarium that morphine does to opium," we might perhaps call *lactucine.* But, unlike morphine, it has no alkaline reaction. It is nearly insoluble in cold, but partly soluble in hot water, from which it is deposited, on cooling, in scales, resembling those of boracic acid. It dissolves in both strong and diluted alcohol, the more completely as the menstruum is more heated. It is insoluble in ether, and is decomposed, not sublimed, by dry heat. Its bitterness disappears when in combination with an alkali, but is restored on the addition of an acid that is capable of neutralising the latter. Lactucarium is reported to possess all the sedative qualities of opium, with the advantage of causing neither constipation nor cerebral congestion.—*Ibid.*

SUDDEN DEATHS AT STRASBOURG.

The causes of death, as ascertained in twenty-six cases of sudden death at Strasbourg, by post-mortem examination, were as follows:—Apoplexy (cerebral hæmorrhage), 1; serous apoplexy, 1; cerebral congestion, 4; cerebral and pulmonary congestion, 1; hæmoptysis, 1; foreign bodies in the bronchi, 2; pulmonary congestion, 13; syncope, 1; perforation of the intestines, 2.—Total, 26.—*London Medical Journal.*

THE BLINDNESS CAUSED BY VITRIOL.

Dr. R. D. Thomson read a communication before the British Association, which was afterwards published in the proceedings of the Philosophical Society of Glasgow, on the nature and cure of blindness produced by the application of the oil of vitriol to the transparent cornea. The operation by which he hoped to remove the blindness was grounded on the following considerations:—"The basis of animal matter, according to the most recent researches of chemists, appears to be a substance termed protein, consisting of $C_{40}$, $H_{31}$, $N_5$, $O_{12}$, which can be readily prepared from albumen, fibrine, &c., by solution in caustic alkali, and precipitation by acetic acid. This substance appears to be a base, and combines with acids. When sulphuric acid is brought in contact with it a fine white substance is formed, which may be obtained in the state of a white powder by careful washing and drying. It may be conveniently produced by triturating the crystalline lens of the eye in a mortar, together with sulphuric acid. This acid is termed *sulpho-proteic*, and its formula is $Pr. + SO_3$."

The conjunctiva contains, as its base, protein, and if sulphuric acid is brought in contact with it, sulpho-proteic acid is formed, and opacity of the conjunctival layer covering the cornea is the result. Dr. R. D. Thomson found, by making a series of experiments upon the eyes of dead animals, that when sulphuric acid was applied to the cornea, a layer of sulpho-proteic acid was produced, which could be removed by means of a sharp-edged knife; and that, even after dissecting off the first layer, a second application of the acid will produce a new layer of sulpho-proteic acid, which may be excised or torn off in a similar manner; and in this way that the whole of the cornea may be successively divided into a series of layers, corresponding, in some degree, with the natural structure of that membrane. This method presents, in short, an excellent mode of demonstrating anatomically the layers of the cornea.

Success having thus far attended the experiments on the dead animals, Dr. Thomson determined to extend the principle to the living eye, for which purpose a dog was obtained, properly secured, and the sulphuric acid rubbed by means of a glass rod over the transparent cornea. White opacity was produced in a few seconds. The dog was left quietly for a few minutes, the eyelids being guarded from the influence of the acid, and when again examined, the cornea presented a white appearance, and was obviously quite opaque. The conjunctiva was then removed by means of a pair of scissors, assisted by a scalpel and forceps, and the denuded cornea was then scraped by means of the scalpel, until it appeared to be deprived of its white opacity. A slight degree of dulness remained, which appeared to have proceeded from the exudation on the surface of the cornea, for in a day or two the perfect transparency of the membrane was restored, and the animal lived for many weeks with complete vision of the eye. The dog belonged to Dr. Krauss, who was present at the experiment, and satisfied himself that vision was thoroughly restored in the eye which had been operated on.

Dr. R. D. Thomson recommends this operation to be performed, when attempted, immediately after the receipt of the injury.—*Provincial Medical Journal.*

*Academy of Sciences, Paris. January 16, 1843.*

CARBONIC ACID EXHALED DURING RESPIRATION.

M. Andral read a memoir on the quantity of carbonic acid exhaled during respiration. The following are the conclusions at which the author arrives:

1. The quantity of carbonic acid exhaled in a given time by the lungs, varies according to the age, sex, and constitution of the individual.

2. In both male and female this quantity varies with the age, and the variation is independent of the weight of the individual.

3. At every period of life, from eight years to advanced old age, the quantity of carbonic acid exhaled from the lungs of the male in a given time is, *cæteris paribus*, always more considerable than that furnished by the female. This difference is particularly well marked between the ages of sixteen and forty, during which period the male exhales nearly twice as much carbonic acid as the female.

4. In the male the quantity gradually increases from eight to thirty years, and it is increased suddenly and in a considerable degree at the period of puberty. After the age of thirty the quantity of carbonic acid begins to decline, and the decrement is greater in proportion as extreme old age approaches; so that, at the latter period, the quantity may fall to what it was at the age of ten years.

5. In the female the exhalation of carbonic acid follows the same law of increment during the period of childhood; but as soon as menstruation commences, the increase of this exhalation is suddenly arrested, and it remains stationary during the period over which the function of menstruation extends. When the woman ceases to menstruate, then the exhalation is suddenly and remarkably increased, and it then declines with old age as in the male.

6. During the whole period of pregnancy the exhalation of carbonic acid is raised to the standard peculiar to women at the critical period of life.

7. In both sexes and at all ages the quantity exhaled is great in proportion to the strength of the constitution and the development of the muscular system. This fact is confirmed by experiments of a different kind, showing that weakness of the constitution produced by disease is attended by diminution in the quantity of carbonic acid exhaled.

The considerable variations now noticed do not depend only, as might be thought, on the differences in the capacity of the chest.

IODIDE OF POTASSIUM IN ATONIC ULCERS.

The utility of the hydriodate of potass in secondary syphilis is pretty generally acknowledged. M. Lisfranc has employed it with success in ulcers of long standing, which have resisted other modes of treatment. A man, sixty-eight years of age, was admitted under his care at the Hôpital de la Pitié, who had for eight years had two obstinate atonic ulcers on his left leg; one of which, five inches in length, extended round the limb for more than half of its circumference; the other was about two inches in diameter. The only topical application used was simple cerate and charpie, but M. Lisfranc prescribed a scruple of the iodide of potassium daily, which quantity he subsequently increased to six grains every six hours. At the end of six weeks the health of the patient had become greatly improved; his flesh generally had acquired firmness; his face was ruddy; and he had even gained fat. The smaller of the two ulcers had completely disappeared in twenty-five days, and scarcely one-tenth of the larger ulcer remained to be healed. In two months the man left the hospital perfectly cured.—*London Lancet.* Jan. 7, 1843.

---

Lactate of Quinine, the claims of which to the notice of practitioners appear to have been known some time ago, has somewhat recently found an advocate in Prince Lucien Bonaparte. Among its advantages over the sulphate, enumerated by the prince, the chief seems to be its greater solubility.—*Ibid.*

---

### VARICOCELE.

M. Vidal de Cassis has published in the "Annales de Chirurgie Francaise," a communication on the radical cure of varicocele by a modified application of the ligature, and on its applicability to the treatment of tumours of the testicle. Although, in general, opposed to operations for the cure of varicosed veins, on account of the great risk of the occurrence of fatal phlebitis, the uncertainty of the results, and the frequency of relapse, M. Vidal did not feel the same repugnance to operations for the cure of variocele, which he regards as a purely local affection. His opinion, on the propriety of operating for the removal of the last-named infirmity, has been considerably strengthened of late, and he has, himself, already operated thirty times.

When varicocele occurs in the persons of old men, who have been long afflicted therewith, the operation should not be performed; as the inconveniences to which the patient is subjected are not sufficient to warrant the proceeding, more especially as at an advanced age it could scarcely be undertaken without involving some risk of a fatal termination. M. Vidal, however, has seen it occur before the fifteenth year, and says that he has very rarely met with a case where it commenced after the thirtieth. Among the reasons alleged by him for operating on this variety of varix, when it occurs in young persons, are: the inconvenience and pain which it causes, the impediments it offers to exercise or moderate exertion, the sterility it induces, and the absence of the varicose diathesis. Patients affected with varicocele, as might be anticipated from their age, are rarely subject to a varicose condition of the veins of the legs, to hæmorrhoids, or any other disease connected with varix. He considers varicocele, as has been already remarked, to be essentially a local affection, arising from excessive exercise of the genital organs, either with women, or onanism, or by other excitement of the testicles. He narrates a case of varicocele following orchitis, in the person of a young man much addicted to women, in illustration of his position: the epididymis of the left side was enlarged and indurated, consequent on a gonorrhœa and orchitis, and the veins of that side were varicose. The diseased state of the veins followed so rapidly the supervention of inflammation of the testicle, that M. Vidal considers himself entitled to call it an acute affection, in opposition to the same disease occurring in old men, which he terms chronic. Varicocele dependent on the causes above mentioned, being essentially local, is not connected with the varicose diathesis to which varix, occurring in another part of the body, may be referred; that is to say, the latter variety is dependent on several causes, seated sometimes at a distance from the part affected.

M. Reynaud, of Toulon, whose mode of operating, somewhat modified, has been adopted by M. Vidal de Cassis, proceeded as follows: He seized the spermatic cord of the diseased side with both hands; he sought for, recognised, separated, and pushed inwards the vas deferens towards the root of the penis; he distinguished it from the vessels and nerves of the testicle by its hardness; then pinching up the scrotum between the thumb and forefinger of the left hand, so as to embrace the spermatic vessels and nerves, he traverses the base of it with a curved needle, armed with a waxed thread. He then releases his hold of the scrotum, brings the two ends of the thread together, and then ties over them a thick but short cylinder of linen, previously placed between the thread and the skin. The ligature must be so tied that it may be loosed or unfastened, in case it should become necessary to diminish the compression exerted on the parts. The only dressing required is the application of lint spread with simple cerate, and a compress. No bandage is necessary. A slight degree of inflammation generally arises, but it lasts but a short time, and two or three days after the operation the ligature may be tightened over a fresh cylinder. If, however, the inflammation be sufficiently extensive and the pain severe, when the ligature is untied, it must not be re-secured until the inflammation has been removed by emollient applications.

The tightening of the ligature, from time to time, causes the gradual division of the soft parts, which cicatrise gradually from the part where the needle entered; by the fifteenth or eighteenth day the spermatic nerves and vessels are cut through, and there remains only the skin undivided; M. Reynaud, then, not to leave a doubt as to the perfect section and obliteration of the vessels of the cord, passes a grooved director, and divides the remaining integument with the bistoury. The wound which results from this is soon healed, and the patients are generally discharged cured in twenty-five days after the operation.

The modifications introduced by M. Vidal, consist in the substitution of a piece of silver wire for the ligature instead of the waxed thread, by which he is enabled to tighten or relax the constriction without undoing the knot, by merely passing the end of a director between the wire and the linen cylinder, and twisting or untwisting it as occasion may require, and the use of a straight instead of a curved needle for the application of the ligature. He also converts the operation into a subcutaneous one by not dividing the integument, when by drawing a little on the ends of the wire, he finds the vessels are divided. If he believes that there is danger of a relapse, he passes two ligatures, about an inch distant from each other, the first of which only is knotted and tightened, the other, placed nearer the testicle, serves as a *ligature d'attente.* If the first ligature produce sufficient inflammation and swelling of the varicose mass below, the other ligature is not tightened, and if the inflammation run very high, it may be removed, and the first one relaxed, but it is as well to retain the *fil d'attente* as long as possible; for if, after the veins have been cut through, and the induration has disappeared, a varicose condition of the cord still continues, or there should be a tendency to relapse, the second ligature may be tightened, and a radical cure be effected.

The same operation has been applied by M. Vidal de Cassis in two cases, to the treatment of varicocele, attended with enlargement of the testicle, and in both instances with complete success. He proposes further to extend its application to the treatment of incurable tumours of the testicle and of other parts, which may be in an analogous condition. In fact, he says, if the ligature does not effect a definitive cure, it may arrest the disease, prevent its ulterior development, and if it be malignant, put an obstacle to its propagation at a distance. At all events it may be preparatory to extirpation, and afford a better chance of success for the latter operation. In the latter case, the spermatic veins alone are not to be tied, but the entire cord must be ligatured; an operation, however, only to be performed in cases where the testicle is much diseased, as in true scirrhus and tubercular degeneration.—*Prov. Med. Journ, Jan.* 24, 1843.

---

*Means of rendering the Preservation and Use of the Nitrate of Silver more certain and more easy.* By Professor Dumeril.

The little cylinders of lunar caustic are generally kept in shops, in a bottle well corked, and separated from each other by linseed, or coriander seeds, to preserve them from the action of the light, air, and moisture. As these cylinders of melted nitrate of silver have not been always moulded at the same degree of fusion, when too much acid has remained in the material, it crystallizes in the mould, by radiating striæ, and unfortunately the cylinders are then friable, very brittle, and necessarily difficult to fix between the sides of a porte-crayon. They must be cut in order to introduce them into the metallic tube, and in doing this they are often broken, and if they are too thin, they break on the least effort. In all cases it is not easy to cut or scrape them, to adapt them to the surfaces on which we wish the caustic to act, whether it be on a single point, or on a line; a flat surface, or the whole extent of a large ulcer. By the little improvement I am about to suggest, I obtain all these advantages. It consists in melting at the fire, or dissolving in pure alcohol, good sealing-wax used by engravers, which contains a good deal of gum lac; holding in a forceps the cylinder of nitrate of silver, I dip it in this solution. The material of the lac applies itself perfectly round the nitrate of silver, and covers it entirely; it adheres to it on all sides, and very strongly; as a varnish unalterable by the air, and whose surface is very smooth, and impenetrable to moisture.

The nitrate of silver, so prepared, can be touched with impunity: it does not stain the fingers; it is very strong, in consequence of its envelope. It resists the pressure of the porte-crayon, which it no longer corrodes. By scraping off some of the covering, as much of the surface may be exposed as is thought necessary.

The great advantage in this mode of preparation is, that I can fix the caustic with firmness, and carry or insinuate it without danger, to any sufficient distance down the throat, or the other cavities.—*Dublin Journal,* 1843, *from Gaz. Med.*

---

DIFFERENCE OF THE PUS-LIKE GLOBULES OF THE BLOOD IN HEALTH FROM THOSE IN DISEASE.

Although the pus-like globules which are found in the blood of patients who are affected with various severe inflammatory and suppurative diseases, are very like the pale globules now so well known as belonging to healthy blood, it often happens that the former globules differ manifestly from the latter. In inflammatory affections the pus-like globules of the blood are generally rather larger, more irregular in size and form, and sometimes more opake, than the pale globules of healthy blood; and the globules occurring in disease are frequently clustered together very remarkably. They are sometimes of a reddish colour, including from one to four blood-discs, rarely five or six, in a very delicate and pale envelope. Besides, in the pus-like globules of the blood of patients labouring under inflammatory disease, the molecules composing the nucleus are mostly surrounded, and often are more or less separated, by a quantity of minutely granular matter, which is either generally less obvious, or even absent, in the pale globules of healthy blood. In a case of great swelling, with purulent deposit, in the leg of a mare, the pus-like globules presented an average diameter of $\frac{1}{2666}$th of an inch, and were nearly as numerous as the red dics; while in the blood of a healthy mare, examined at the same time, for comparison, the pus-like globules were by no means so plentiful, and they almost all ranged between $\frac{1}{3500}$th and $\frac{1}{2900}$th of an inch.—*Mr. Gulliver, Lond. and Edin. Phil. Mag.*, Sept. 1842.

---

MODE OF FORMATION OF RED GLOBULES.

Dr. Remak, of Berlin, has been able to distinguish these bodies in the blood of an embryo chick in the third week of artificial incubation, as well as in the embryo pig an inch in length, in which last he finds they are from four to six times as large as in the full grown animal. In the chick they are of different shapes,—rounded, pyriform, and pediculated, or elongated, and with a nucleus at either end, united by a minute prolongation. This last condition has given rise to a notion that the reproduction of the globules takes place by a procedure analogous to fissiparous generation. Dr. Remak had recourse to the following experiment to ascertain in what way the red globules were reproduced, and the facts that he observed throw some light on the process:—A horse was bled for several successive days, as much as 30lbs. of blood being taken from him on the first day. At that time the red globules were very abundant, while only a few colourless globules were present. Next day the latter were numerous, and mostly enlarged, having in their interior one or more globules of a pale-red colour surrounded by small granules. On the succeeding days these globules deepened in colour, and, correspondingly, the accompanying granules disappeared. On the fourth day it became evident that red globules, similar to those existing in the blood in an independent state, had been formed within the larger colourless corpuscules, and set free by the bursting and disappearance of the latter. Similar phenomena presented themselves in similar observations on the blood of the frog and the human subject. In man, between the fourth and eighth day, after the loss of a large quantity of blood, and notwithstanding the presence of typhoid and inflammatory diseases, the reproduction of the red globules goes on in the same way, as proved by the discovery of a proportionable quantity of the outer films of the colourless globules in the clot.—*Lond. Lancet, from Med. Zeitung*, No. 27.

